# Joint Self-Calibration of Receiver Geometry, Timing, and Target Positions for Multistatic Radar Autofocus

**DOI:** 10.3390/s26154954

**Published:** 2026-08-05

**Authors:** Anthony J. Weiss, Guy Eliyahu, Amnon Menashe Maor, Ezra Zamir, Oran Richman

**Affiliations:** 1 School of Electrical Engineering and Computers, Tel Aviv University, Tel Aviv 6139001, Israel; 2 Elbit Systems Land & C4I Ltd., Netanya 4250712, Israelamnonmenashe.maor@elbitsystems.com (A.M.M.); ezra.zamir@elbitsystems.com (E.Z.); oranrichman@gmail.com (O.R.)

**Keywords:** multistatic radar, autofocus, self-calibration, sensor network calibration, time synchronization, near-field imaging, matched filtering, maximum a posteriori estimation, identifiability, free network adjustment, CLEAN deconvolution, transmitter localization, multilateration

## Abstract

Near-field multistatic radar imaging assumes that the transmitter, receiver, and target positions, as well as the receiver time references, are known exactly. In practice they are known only approximately: receiver positions and clocks carry small survey and synchronization errors, and target locations used to initialize or refine an image are themselves approximate. This paper develops a joint self-calibration framework that estimates small corrections to receiver positions, receiver clock biases, and target positions from the same bistatic echo delays used for imaging, and ties the correction directly to image sharpness rather than to parameter accuracy alone. We derive the linearized observation model relating delay residuals to these corrections, and give a regularized (maximum a posteriori) weighted least-squares estimator that explicitly separates measurement noise from prior parameter uncertainty. We characterize the identifiability of this estimator progressively, from a single anchor (the transmitter alone, which leaves an exact three-dimensional rotational null space) to two anchors (transmitter plus one additional point, which reduces the null space to a one-parameter rotation about a fixed axis) to three anchors (transmitter, one target, and one receiver, in general position, which removes the continuous ambiguity entirely). We additionally treat the dual problem of localizing an unknown transmitter from a small number of exactly known anchors—receivers, targets, or time samples of a single moving platform—and show that collinear or coplanar anchor geometries leave an exact, uncorrectable continuous or discrete ambiguity, respectively, regardless of how many such anchors are used, with the coplanar case notably invisible to a standard rank or conditioning check. We then reformulate the calibration objective directly in terms of coherent multistatic image sharpness, evaluated using the matched-filter score already used for image formation, and propose a two-stage algorithm: a coarse linear delay-residual solve followed by phase-coherent sharpness refinement. Numerical experiments verify the predicted identifiability transitions via the singular value spectrum of the linearized system, demonstrate quadratic convergence of the proposed estimator, verify the transmitter-localization ambiguity structure—including an exact mirror-twin solution for coplanar anchors, reproducing all range measurements to floating-point precision—and demonstrate the effect of self-calibration on a simulated multistatic image of an extended (eagle-shaped) target, including the incremental effect of bandwidth, aperture/frequency windowing, and CLEAN deconvolution on the recognizability of the resulting image, as well as on the resolvability of multiple simultaneous discrete targets (two instances of the same target). We relate this formulation to, and distinguish it from, the existing literature on time-of-arrival sensor network self-calibration and on joint target-localization/clock-bias estimation, which largely target single moving targets, anchor-free minimal-data solvability, or localization accuracy rather than multistatic image focus.

## 1. Introduction

Near-field multistatic radar imaging forms a coherent image of a scene by combining echoes observed at several spatially distributed receivers illuminated by a common transmitter. Image formation—whether by direct matched filtering, backprojection, or an accelerated approximation such as receiver-domain decomposition—relies on an accurate geometric model: the transmitter position, the receiver positions, the receiver time references, and, when used for initialization or refinement, approximate target positions. In practice, none of these quantities is known exactly. Receiver positions carry residual survey or GPS error; receiver clocks carry synchronization offsets even after calibration; and target positions, when available at all, are themselves estimates from a previous processing stage. Left uncorrected, these errors defocus the resulting image in the same way that uncompensated platform motion defocuses a synthetic aperture radar image.

Classical autofocus techniques, such as phase gradient autofocus [1], correct this kind of defocusing without an explicit geometric model: they estimate and remove an unknown phase error directly from the data. This is powerful when the error has no assumed structure, but it discards information that is available in a network of receivers with known—if imperfect—geometry: every receiver-position error and every clock bias affects a specific, physically parameterized subset of the observed delays, and different targets seen from different subapertures constrain those same parameters jointly. This paper develops a self-calibration framework that exploits this structure directly, ties the resulting correction to coherent image sharpness rather than to parameter accuracy alone, and gives conditions under which the resulting estimation problem is well posed.

### 1.1. Related Work

Joint estimation of sensor geometry, timing, and target position from range or time-of-arrival (TOA) measurements has been studied extensively outside the radar-imaging literature, primarily in the sensor network self-calibration and source localization communities.

A substantial body of work by Åström, Kuang, Burgess, and coauthors formulates TOA network self-calibration as an algebraic geometry problem: given only pairwise transmitter–receiver distances, determine for which configurations the transmitter and receiver positions are uniquely recoverable, and construct minimal, non-iterative solvers for those configurations [2,3]. This line of work gives a rigorous characterization of well-posedness, but it targets the anchor-free, near-exact-data regime—the goal is to recover an entire network from scratch with the fewest possible measurements—rather than refining an already approximately known network using redundant, noisy measurements and informative priors, which is the regime of interest here.

Closer to the present problem, several recent papers jointly estimate a target position together with sensor position and/or clock-bias corrections. Jia, Ke, Liu, Ho, and Su [4] jointly localize a constant-velocity moving target and self-calibrate sensor position and synchronization offsets from a sequence of range and angle measurements, and derive Cramér-Rao bounds showing that at least two sensors are required for self-calibration. Ke and Ho [5] address a related pseudo-range localization problem without synchronization using rigid-body receiver constraints. Zhang, Shi, Wang, Xu, and Pang [6] jointly estimate a stationary target position and per-platform clock bias in mobile bistatic sonar using measurements collected over multiple time steps. Yousefi, Chang, and Champagne [7] jointly estimate target position and receiver clock biases from TOA measurements at multiple antenna receivers. Sagduyu, Davaslioglu, Erpek, Kompella, Anderson, and Ashdown [8] treat receiver clock bias explicitly in a multistatic integrated sensing-and-communications setting, showing that a common (single) offset across receivers is jointly identifiable with target position given a sufficient number of receivers.

Each of these works estimates target position and calibration parameters as an end in itself, evaluated against parameter accuracy or the CRB. None of them (i) treats multiple simultaneous stationary targets as joint calibration references, (ii) explicitly separates the roles of measurement noise and prior parameter uncertainty in a MAP formulation, or (iii) ties the calibration objective to coherent image sharpness of a multistatic matched-filter image rather than to estimation accuracy of the geometric parameters themselves. The present paper addresses all three points, and in doing so connects this literature to image-domain autofocus for the first time in this form, to the best of our knowledge. To state the novelty as concretely as possible, the prior TOA self-calibration and joint localization/clock-bias literature answers “how accurately can we recover sensor and target parameters,” whereas the single new element introduced here is to answer instead “how much does that recovered geometry improve the coherent multistatic image,” by reformulating the same class of estimators (Section 2.2, Section 2.3 and Section 2.4) as a two-stage procedure whose second stage optimizes image sharpness *J* directly (Section 2.7) rather than stopping at the parameter estimate itself—together with the progressive anchor-count identifiability analysis (Section 2.5) that this image-domain reformulation requires to be well posed in the first place.

A second, complementary body of work approaches sensor calibration from a data-driven rather than a model-based direction. In radar–camera extrinsic calibration, for instance, deep learning feature matching and nonlinear refinement have been used to estimate the extrinsic transform from raw radar and camera data without an explicit geometric error model [9,10]. Such methods can be attractive when the sensing modalities are heterogeneous enough (e.g., radar range–Doppler–angle maps versus camera imagery) that a shared first-principles observation model of the kind used here is not readily available, and they can absorb calibration-relevant effects (feature-extraction noise, modest model mismatch) that are difficult to specify analytically. The present, model-based approach trades that flexibility for three properties that a learned calibration typically does not provide by construction: interpretability of every estimated correction in physical units (position, clock bias), no requirement for labeled training data or a representative training deployment, and an explicit, provable identifiability characterization (Section 2.5) of exactly which corrections are and are not recoverable from a given anchor configuration, rather than an empirical accuracy figure on a specific test set. The corresponding cost is that the present method depends more heavily than a learned calibration would on accurate initialization (Section 2.2), on the anchor geometry satisfying Propositions 1 and 2, and on the bistatic delay model (2) and (3) being an adequate description of the actual propagation and detection process—an assumption revisited in Section 5 alongside other departures from the idealized measurement model considered here.

The identifiability analysis given in Section 2.5 is a special case of the classical free network adjustment (or datum defect) problem in geodesy, in which a network of range or distance observations determines relative geometry only up to a residual rigid-motion ambiguity that must be removed by external constraints [11]. That literature provides the general machinery; Section 2.5 specializes it to the single-transmitter multistatic radar case, and additionally gives the intermediate one-axis ambiguity that survives with only two anchors, which is a step not needed in the classical free network setting.

Finally, the image-domain deconvolution used in Section 4 to sharpen the reconstructed multistatic image is an application of the CLEAN algorithm, originally developed for radio-interferometric imaging with sparse, incomplete aperture (visibility) coverage [12]. To the best of our knowledge, CLEAN has not previously been combined with geometry/timing self-calibration of the kind developed here for multistatic radar.

### 1.2. Contributions

The main contributions of this paper are as follows:

(1) A linearized joint observation model relating bistatic delay residuals to small corrections in receiver position, receiver clock bias, and target position, with an explicit, separate accounting of measurement noise and prior parameter uncertainty.
(2) A progressive characterization of the gauge freedom of this model, from one anchor (the transmitter alone: an exact three-dimensional rotational null space) to two anchors (transmitter plus one additional point: a one-parameter axial rotation ambiguity) to three anchors (transmitter, one target, one receiver, in general position: the continuous ambiguity is fully removed).
(3) A reformulation of the calibration objective in terms of coherent multistatic image sharpness, evaluated using the same matched-filter score used for image formation, rather than parameter estimation accuracy.
(4) A two-stage estimation algorithm—coarse linearized delay-residual correction followed by phase-coherent sharpness refinement—together with a discussion of its relationship to the prior TOA self-calibration and joint localization/synchronization literature.
(5) A treatment of the dual problem—localizing an unknown transmitter from a small number of exactly known anchors, such as receivers, targets, or time samples of a single moving reference platform—including a characterization of the collinear and coplanar anchor-geometry degeneracies under which the identifiability rank test alone is insufficient to detect a surviving ambiguity.
(6) Numerical verification of the predicted identifiability transitions and estimator convergence, and a demonstration of the effect of self-calibration, wideband/windowed sidelobe suppression, and CLEAN deconvolution on the recognizability of a simulated multistatic image of an extended target. We stress that only the self-calibration estimator of contributions (1)–(4) is the paper’s core contribution: CLEAN is applied afterward, as an independent, off-the-shelf image-domain post-processing step on the already-self-calibrated image, not as an integrated part of the calibration objective itself. Section 4.6 reports its effect in isolation for exactly this reason.

### 1.3. Paper Organization

Section 2 develops the observation model, the MAP estimator, the progressive identifiability analysis, the dual transmitter-localization problem, and the image-sharpness objective. Section 3 presents the two-stage estimation algorithm. Section 4 reports numerical experiments verifying the identifiability analysis and estimator convergence, and demonstrating the effect of self-calibration and image-domain sidelobe suppression on a simulated extended-target image. Section 5 discusses the findings and their implications. Section 6 concludes and summarizes limitations.

## 2. Mathematical Model

### 2.1. Geometry and Signal Model

**Figure 1:**
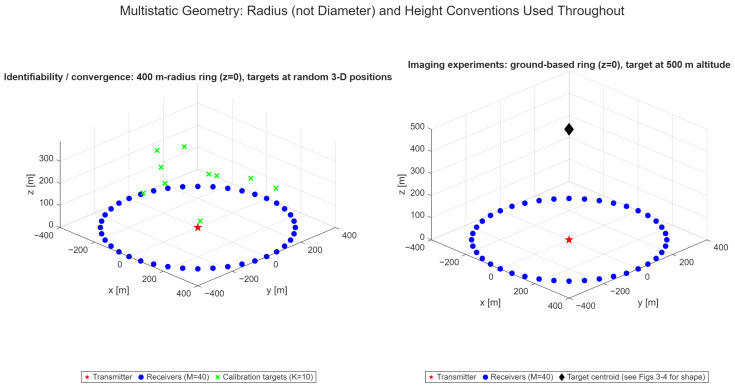
The two experiment geometries used throughout, both with M=40 receivers on a ring of radius 400 m. Left: The general geometry used for the identifiability (Section 4.1) and convergence (Section 4.2) experiments, with K=10 calibration targets at three-dimensional positions with range and height each drawn uniformly over [100,400] m—not confined to the receivers’ z=0 plane or to any other common height. For illustrative clarity here, the K=10 targets are spread evenly in azimuth around the ring; the actual identifiability/convergence experiments instead draw all three coordinates i.i.d. uniformly, which can (and, for the specific random draw underlying the reported numbers, does) cluster the targets within a narrower angular range purely by chance—a property of genuine random sampling, not a schematic-only choice. Right: The look-up geometry used for the imaging experiments (Section 4.4, Section 4.5, Section 4.6 and Section 4.7), with the transmitter and all M=40 receivers on the ground (z=0, consistent with ground-based sensors) and the real (imaged) target elevated to a representative 500 m flying altitude, shown here only as a centroid marker since the target’s actual ∼1 m internal structure is far too small to see at this 400–500 m scale (its structure is instead shown at full imaging resolution in later figures).

Consider a single transmitter at exactly known position $p_{tx}$, *K* calibration targets with nominal (approximately known) positions $p_{k}^{0}$, k=1,…,K, and *M* receivers with nominal positions $q_{m}^{0}$, m=1,…,M. Figure 1 shows the two representative geometries used throughout the numerical results (Section 4): a general geometry with calibration targets at unconstrained three-dimensional positions (used for the identifiability and convergence experiments), and a look-up imaging geometry with the transmitter/receiver ring on the ground and the (real, imaged) target elevated above it (used for the imaging experiments, consistent with an airborne target such as a bird observed by ground-based sensors)—in both cases the receiver ring’s 400 m figure is a radius, not a diameter. The true calibration-target and receiver positions are

(1) $$p_{k}=p_{k}^{0}+\delta p_{k},\;q_{m}=q_{m}^{0}+\delta q_{m},$$

with $\delta p_{k}$, $\delta q_{m}$ being small corrections.

All times in this paper are referenced to the clock of a single designated receiver, taken without loss of generality to be receiver 1, whose clock defines the time origin ($\delta t_{1}\equiv 0$); the transmitter’s own clock is not assumed exact against this origin. Every other receiver m=2,…,M carries an unknown, small clock bias $\delta t_{m}$ relative to receiver 1’s clock, and the transmitter carries an unknown, small emission-time offset $\delta t_{tx}$ relative to the same reference, common to every delay measurement from a given transmission. For calibration target *k* observed at receiver *m*, the true propagation delay from the transmitter through the target to the receiver, expressed in receiver-1 time, is

(2) $$\tau_{km}=\frac{∥p_{k}-p_{tx}∥+∥q_{m}-p_{k}∥}{c},$$

where *c* is the propagation speed. What is actually measured at receiver *m* is not this propagation delay directly, but the time of arrival of the echo of calibration target *k*, read on receiver *m*’s own clock; the resulting time-of-arrival estimate ${\hat{\tau }}_{km}$, extracted from the matched-filter or correlation peak, is

(3) $${\hat{\tau }}_{km}=\delta t_{tx}+\tau_{km}+\delta t_{m}+n_{km},\;\delta t_{1}\equiv 0,$$

where $n_{km}$ is measurement noise, distinct from the parameter uncertainty in (1). Its variance is set by the delay-estimation accuracy of the matched filter for the transmitted waveform and the per-pair signal-to-noise ratio, and may therefore differ across receiver–calibration-target pairs. Note that $\delta t_{tx}$ enters every measurement from a given transmission identically, while $\delta t_{m}$ is specific to receiver *m*; the two are therefore distinguishable whenever M≥2, exactly as in the transmitter-referenced parametrization used implicitly in earlier treatments of this model—the choice of time origin is a relabeling of the same clock-bias subspace, not a change to it, as we verify explicitly below.

### 2.2. Linearized Observation Model and MAP Estimator

Let $\tau_{km}^{0}=\tau_{km}\left(p_{k}^{0},q_{m}^{0}\right)$ denote the delay predicted from the nominal geometry and $\delta {\hat{\tau }}_{km}={\hat{\tau }}_{km}-\tau_{km}^{0}$ the observed residual. Since all corrections are assumed small, a first-order expansion of (2) and (3) gives

(4) $$\delta {\hat{\tau }}_{km}\approx -\frac{1}{c}{\left({\hat{u}}_{k,tx}+{\hat{u}}_{k,m}\right)}^{T}\delta p_{k}\;+\;\frac{1}{c}{\hat{u}}_{k,m}^{T}\delta q_{m}\;+\;\delta t_{tx}\;+\;\delta t_{m}\;+\;n_{km},$$

where ${\hat{u}}_{k,tx}$ and ${\hat{u}}_{k,m}$ are unit vectors from calibration target *k* toward the transmitter and toward receiver *m*, respectively (both signs verified against a finite-difference check of the exact delay model (2)), and $\delta t_{1}\equiv 0$. Stacking all corrections into

(5) $$x={\left[\delta p_{1}^{T},\ldots ,\delta p_{K}^{T},\;\delta q_{1}^{T},\ldots ,\delta q_{M}^{T},\;\delta t_{tx},\delta t_{2},\ldots ,\delta t_{M}\right]}^{T}\in R^{3K+4M}$$

(for the 3-D geometry), every valid (k,m) pair contributes one row of a sparse linear system

(6) b=Ax+n,

where row km of A has nonzero entries only in the blocks for calibration target *k* and receiver *m*. The parameter count above is unchanged from a transmitter-referenced convention (*M* clock unknowns either way): one degree of freedom moves from “receiver 1’s bias relative to the transmitter” to “the transmitter’s offset relative to receiver 1,” and $\delta t_{m}$ for m≥2 is reinterpreted relative to the new origin. The column space of A, the rank of $A^{T}WA$, and therefore every identifiability statement in Section 2.5 are unaffected: this is a change of basis on the clock-bias block of x, not a change to the linear system itself. Given a prior $x∼N(0,\Sigma_{0})$ and noise covariance $W^{-1}=\mathrm{Cov}\left(n\right)$, the MAP estimate is

(7) $$\hat{x}=\arg\min_{x}{\;∥b-Ax∥}_{W}^{2}+{∥x∥}_{\Sigma_{0}^{-1}}^{2}={\left(A^{T}WA+\Sigma_{0}^{-1}\right)}^{-1}A^{T}Wb.$$

Because (4) is only a first-order approximation, (7) is applied iteratively (Gauss–Newton): the nominal geometry is updated by $\hat{x}$, $\tau_{km}^{0}$ and A are recomputed at the new point, and the process repeats until $∥\hat{x}∥$ falls below a convergence threshold.

#### Where the Calibration Targets Come from, and How Coarse Their Initial Positions May Be

The framework above assumes *K* calibration targets with nominal positions $p_{k}^{0}$, but is agnostic to their provenance: they may be dedicated, surveyed reference reflectors placed for calibration purposes, a cooperative transponder or corner-reflector-equipped calibration platform (e.g., a UAV, as in Section 2.6) whose position is independently reported, or simply a small number of scene targets whose approximate positions are read off a coarse, uncorrected image formed with the nominal (uncalibrated) geometry itself—in the last case, $p_{k}^{0}$ is not external side information at all, but the output of a preceding, cruder processing stage, and Stage 1 (Section 3) refines both the geometry and, implicitly, those target position estimates together. This third option is the most practically important, since it removes any dependence on an external positioning system, and it is compatible with the estimator as written: nothing in (4)–(7) distinguishes a nominal position obtained from a coarse image peak from one obtained by survey.

What limits how coarse $p_{k}^{0}$, $q_{m}^{0}$ may be is exactly the validity of the first-order expansion (4): the linearization error in the bistatic delay (2) is second-order in the correction, i.e., $O(∥\delta p_{k}{∥}^{2}/R)$ relative to the leading term, where *R* is the relevant transmitter–target or target–receiver range. For the geometry used throughout Section 4 (*R* of order $10^{2}$–$10^{3}$ m), an initial error of a few meters keeps this relative curvature term several orders of magnitude below the sub-nanosecond delay precision of interest, which is why the Gauss–Newton iteration of Section 4.2 converges quadratically from RMS initial errors of 0.66–0.74 m; an initial error approaching a significant fraction of *R* itself, by contrast, would leave the linearization error comparable to the signal of interest and require either a coarser, nonlinearized search (e.g., a direct grid search over Q(·), analogous to the acquisition step used elsewhere in this line of work for locating a target before fine tracking begins) or a trust-region/damped Gauss–Newton variant before (7) is applied directly. We have not characterized the exact breakdown radius numerically in this paper, since every experiment in Section 4 uses errors in the regime where quadratic convergence is observed; doing so systematically—i.e., mapping the basin of quadratic convergence as a function of $∥\delta p_{k}∥/R$ and $∥\delta q_{m}∥/R$—is a natural and tractable extension we leave for future work, since it requires only repeated re-runs of the existing Stage 1 iteration from increasingly perturbed starting points, not a new estimator.

The distinction between W and $\Sigma_{0}^{-1}$ in (7) is deliberate: only $\Sigma_{0}$ describes belief about the nominal geometry before any measurement is taken, while W describes the precision of the delay measurements themselves. Reducing measurement noise (larger W) improves the estimate within whatever subspace of x the data can resolve; it has no effect on directions of x that are structurally unobservable from (6), which are governed entirely by $\Sigma_{0}$ instead. Section 2.5 identifies exactly such directions, and shows how they shrink as anchors are added.

### 2.3. Summary of Notation

Several similarly named quantities have been introduced above (transmitter timing offset, receiver clock bias, delay residuals, and the various position corrections); Table 1 collects all of them in one place for reference.

### 2.4. Cramér–Rao Lower Bound

To assess how close the estimator (7) comes to the best achievable accuracy, we compare it against the Cramér–Rao lower bound (CRLB) for x under the measurement model (6). Two variants are relevant, corresponding to whether the prior $x∼N(0,\Sigma_{0})$ is treated as genuine side information or purely as a regularizer used to remove the gauge freedom of Section 2.5.

#### 2.4.1. Unbiased (Data-Only) CRLB

Treating x as a deterministic unknown observed through b=Ax+n, $n∼N(0,W^{-1})$, the Fisher information matrix is

(8) $$J=A^{T}WA,$$

and, whenever J is nonsingular, the CRLB for any unbiased estimator of $x_{i}$ (the *i*-th entry of x) is $\mathrm{Var}\left({\hat{x}}_{i}\right)\geq {\left[J^{-1}\right]}_{ii}$. By Propositions 1 and 2, J is exactly singular with fewer than three non-transmitter anchors (in 3-D), so the unbiased CRLB is only meaningful once enough anchors are fixed to remove the rotational null space; with those anchors’ corrections held at zero (removed from x and the corresponding columns of A), J is generically nonsingular and (8) applies directly to the remaining, identifiable sub-vector of x.

#### 2.4.2. Bayesian (MAP) CRLB

When the prior is instead used as genuine information about the nominal geometry—the setting of (7)—the relevant bound is the Bayesian CRLB (van Trees), obtained by adding the prior’s information to (8):

(9) $$J_{\mathrm{MAP}}=A^{T}WA+\Sigma_{0}^{-1},\;\mathrm{Var}\left({\hat{x}}_{i}\right)\geq {\left[J_{\mathrm{MAP}}^{-1}\right]}_{ii}.$$

Because $\Sigma_{0}^{-1}$ is positive definite, $J_{\mathrm{MAP}}$ is nonsingular even in regimes where J alone is not, so (9) remains well defined at one or two anchors as well; in that regime, however, the bound in the previously unobservable directions is dominated entirely by $\Sigma_{0}$ rather than by the measurements, and should be interpreted as a statement about prior informativeness rather than about what the data contribute. We note that $\hat{x}=J_{\mathrm{MAP}}^{-1}A^{T}Wb$ in (7) is exactly the linear estimator that attains (9) with equality for a linear Gaussian model, so any gap observed numerically between the achieved RMS error and (9) in Section 4.3 reflects the residual nonlinearity of (2) and (3) (i.e., Gauss–Newton re-linearization error) rather than a suboptimal choice of estimator.

#### 2.4.3. Sensitivity to the Gaussian Noise Assumption

The estimator (7) and both CRLB variants above assume $n_{km}$ in (3) is Gaussian. Matched-filter delay estimates are well approximated as Gaussian at a moderate-to-high per-pair SNR, but this can break down under a low SNR or multipath: the matched-filter output can develop a heavier-tailed error distribution (occasional large outliers from noise-induced false peaks) at a low SNR, and multipath can bias the peak location in a direction set by the specific propagation environment rather than contribute symmetric, zero-mean error. Two consequences follow. First, because (7) is a weighted least-squares estimator, it remains the minimum-variance linear estimator regardless of the true noise distribution, by the Gauss–Markov theorem, provided $W^{-1}$ is set to the actual (possibly non-Gaussian) measurement covariance rather than an assumed one; what is lost under non-Gaussian noise is not consistency of (7) itself but the optimality guarantee of the CRLB comparison in Section 2.4, and the practical accuracy of (7) degrades gracefully with the tails of the noise rather than catastrophically, provided outliers are a minority of the measurements. Second, a small fraction of grossly outlying ${\hat{\tau }}_{km}$ (e.g., a false matched-filter peak from a strong multipath return) can still disproportionately distort the least-squares solution, since (7) weights every residual quadratically; a standard, low-cost remedy compatible with the estimator as written is to replace the Gaussian weighting in (7) with an iteratively re-weighted least-squares (IRLS) scheme using a robust loss (e.g., Huber or Tukey) on the delay residual, which reduces to (7) exactly when all residuals are well-described by $W^{-1}$ and down-weights individual measurements automatically otherwise. We have not implemented or evaluated this variant numerically in the present paper—every experiment in Section 4 uses simulated Gaussian delay noise—so quantifying the estimator’s actual degradation under multipath-induced or low-SNR non-Gaussian errors, and validating the IRLS remedy sketched above, is left for future work.

### 2.5. Identifiability and Gauge Freedom: From One Anchor to Three

We now characterize the gauge freedom of (6) progressively, as successive points in the scene are assumed exactly known (anchored) rather than subject to a correction. Throughout, $p_{tx}$ is always exactly known, since it is the coordinate origin of the whole problem; we refer to the number of additional exactly known points as the number of anchors.

#### 2.5.1. One Anchor: The Transmitter Alone

**Proposition** **1.** *Let $p_{tx}$ be exactly known and let R be any rotation (orthogonal linear map with determinant +1) about $p_{tx}$. Applying R simultaneously to every true target and receiver position, $p_{k}\mapsto p_{tx}+R\left(p_{k}-p_{tx}\right)$ and $q_{m}\mapsto p_{tx}+R\left(q_{m}-p_{tx}\right)$ for all k,m, leaves every bistatic delay $\tau_{km}$ in (2) exactly unchanged.*

**Proof.**  Rotation about $p_{tx}$ preserves distance to $p_{tx}$, so $∥p_{k}-p_{tx}∥$ is unchanged for every *k*. Since *R* is orthogonal and applied identically to $p_{k}$ and $q_{m}$, it preserves their mutual separation: $∥R\left(q_{m}-p_{tx}\right)-R\left(p_{k}-p_{tx}\right)∥=∥R\left(q_{m}-p_{k}\right)∥=∥q_{m}-p_{k}∥$. Both terms of (2) are therefore unchanged for every (k,m). □

With only the transmitter anchored, the sensitivity of $\left\{\tau_{km}\right\}$ to the true positions $\left\{p_{k},q_{m}\right\}$ therefore has an exact, three-dimensional (in 3-D; one-dimensional in 2-D) null space, present regardless of the number, accuracy, or redundancy of the delay measurements: it is a rank deficiency of the underlying model, not a consequence of noise. In the terminology of free network adjustment, this is a datum defect [11]: the single anchor $p_{tx}$ fixes translation relative to itself trivially (all positions are already parameterized relative to it) but does not fix orientation.

#### 2.5.2. Two Anchors: Transmitter Plus One Additional Point

A second anchor—one additional exactly known target or receiver position—does not eliminate the rotational freedom of Proposition 1, but it substantially constrains it.

**Corollary** **1.** *In addition to $p_{tx}$, let one further point $p_{a}$ (a target or a receiver) be known exactly, with $p_{a}\neq p_{tx}$. Then the three-dimensional rotational null space of Proposition 1 reduces to the one-parameter subgroup of rotations about the axis through $p_{tx}$ and $p_{a}$: every such axial rotation, at any angle, still leaves every $\tau_{km}$ unchanged, but no other rotation about $p_{tx}$ does.*

**Proof.**  Every rotation considered fixes $p_{tx}$ by definition. A nontrivial rotation about $p_{tx}$ fixes a second point $p_{a}\neq p_{tx}$ if and only if $p_{a}$ lies on the rotation axis, since points off the axis move along a circle of nonzero radius under a nontrivial rotation. There is exactly one line through $p_{tx}$ and $p_{a}$, so the set of rotations fixing both points is precisely the one-parameter family of rotations (at any angle) about that line; by Proposition 1, every member of this family leaves every $\tau_{km}$ unchanged. □

Corollary 1 is the reason a single additional anchor, while helpful, is not sufficient in general: any residual ambiguity is confined to rotation about one specific, known axis, but that one-parameter freedom remains exactly unobservable from delay measurements alone, independent of their number or precision, for the same structural reason as in Proposition 1.

#### 2.5.3. Three Anchors: Transmitter, One Target, One Receiver

**Proposition** **2.** *If, in addition to $p_{tx}$, one target position $p_{k_{0}}$ and one receiver position $q_{m_{0}}$ are known exactly, and $p_{tx}$, $p_{k_{0}}$, $q_{m_{0}}$ are non-collinear, then the rotational null space is eliminated entirely: the only rotation about $p_{tx}$ that fixes both $p_{k_{0}}$ and $q_{m_{0}}$ is the identity.*

**Proof.**  By Corollary 1 applied with $p_{a}=p_{k_{0}}$, any rotation fixing both $p_{tx}$ and $p_{k_{0}}$ is a rotation about the axis through them (or the identity). For this rotation to also fix $q_{m_{0}}$, either the rotation angle must be zero, or $q_{m_{0}}$ must lie on the same axis, which is excluded by the non-collinearity assumption. Hence only the identity rotation satisfies all three constraints. □

The progression from Proposition 1 (three rotational degrees of freedom unobservable) to Corollary 1 (one degree of freedom unobservable) to Proposition 2 (zero degrees of freedom unobservable, up to a discrete reflection) shows precisely how much each additional anchor is worth: the first anchor beyond the transmitter removes two of the three rotational degrees of freedom; the second removes the last one. This is verified numerically in Section 4.1 via the singular value spectrum of the linearized system A at each anchor count.

A residual discrete reflection ambiguity remains after Proposition 2, but in the regime considered here—local, iterative refinement of an already accurate nominal geometry—it is not of practical concern: Gauss–Newton iteration from a good initial point does not migrate to the reflected solution, which is generically far away in parameter space. It would only be relevant if the estimator were initialized from scratch with no informative nominal geometry, which is not the setting of this paper.

In practice, well-posedness should be checked directly on the realized geometry and receiver–target association, not assumed from Propositions 1 and 2 alone: the singular value decomposition of $A^{T}WA$ should be inspected for (i) near-zero singular values beyond the expected count, indicating unexpected structural degeneracy (e.g., near-collinear anchors, or a receiver observing targets confined to a narrow angular sector), and (ii) the condition number restricted to the complement of the null space, which governs how measurement noise is amplified in the identifiable directions even where the rank is full.

### 2.6. The Dual Problem: Localizing an Unknown Transmitter from Known Anchors

Section 2.5 treated $p_{tx}$ as the single exactly known point anchoring an otherwise uncertain receiver–target constellation. The dual question is equally relevant in practice: if the transmitter position is itself unknown, can it be recovered using a small number of exactly known anchor points—which may be receivers, targets, or samples of a single moving platform (e.g., a calibration UAV) observed over time? This dual problem has a fundamentally different ambiguity structure from Propositions 1 and 2: because only the single point $p_{tx}$ is unknown against an already-rigid, fully known anchor set, there is no rigid-body rotation available to the estimator, and the classical continuous rotational gauge freedom of Proposition 1 does not arise. What remains is classical multilateration geometry, and its identifiability depends on two separate factors: whether the transmitter’s emission time is known, and whether the anchors are geometrically non-degenerate.

Let $\left\{a_{1},\ldots ,a_{K}\right\}$ denote *K* exactly known anchor positions (receivers, targets, or time samples of a single moving anchor with a time-invariant nuisance delay). If each anchor’s exact one-way or round-trip path to/from every other known point in the geometry has already been removed from the corresponding delay measurement ${\hat{\tau }}_{k}$ (as in Section 2.2), the residual observation depends on $p_{tx}$ only through $∥a_{k}-p_{tx}∥$. This is the same range- or range-difference-based localization structure studied in the TOA/TDOA sensor network self-calibration literature reviewed in Section 1.1 [2,3], specialized here to a single unknown point against an already-anchored network, which yields the sharper, closed-form characterization below.

**Proposition** **3.** *If the transmitter’s emission time $t_{0}$ is known, then K≥3 non-collinear anchors determine $p_{tx}$ up to at most a two-fold reflection ambiguity through the plane containing the anchors; a (K+1)-th anchor not lying in that plane removes the ambiguity entirely.*

**Proof.**  Each anchor $a_{k}$ with known range $r_{k}=∥a_{k}-p_{tx}∥$ constrains $p_{tx}$ to a sphere centered at $a_{k}$. Three spheres centered at non-collinear points intersect at at most two points, symmetric about the plane through the three centers (the classical trilateration result); a fourth sphere centered off that plane is satisfied by at most one of the two, resolving the ambiguity. □

**Proposition** **4.** *If $t_{0}$ is unknown, differencing the delay observed at anchor $a_{k}$ against a reference anchor $a_{1}$ eliminates $t_{0}$ (and, if the anchors are receivers, any receiver-independent nuisance delay common to the differenced pair) and yields a hyperboloid constraint on $p_{tx}$ with foci $a_{1},a_{k}$. At least K≥4 non-coplanar anchors are required for a discrete solution; K=3 anchors leave a one-parameter family of candidate positions undetermined regardless of measurement accuracy.*

**Proof.**  Differencing removes one equation’s worth of information relative to Proposition 3 (the common $t_{0}$), so *K* anchors yield only K−1 independent constraints on the three components of $p_{tx}$; three constraints (K=4) are needed for a locally full-rank (rank-3) system, matching the classical requirement that TDOA-based geolocation with an unsynchronized source needs one more reference point than the synchronized (TOA) case, exactly as an unsynchronized GPS receiver requires a fourth satellite. This is verified numerically in Section 4.8. □

**Corollary** **2.** *The anchor count alone does not guarantee identifiability: if all anchors are collinear, a continuous one-parameter ambiguity (rotation about the anchor line) persists regardless of how many collinear anchors are used, exactly as in Corollary 1; if all anchors are coplanar but not collinear, a discrete two-fold reflection ambiguity persists regardless of anchor count, as in Proposition 3. Both degeneracies are broken only by at least one anchor genuinely outside the collinear line or coplanar plane, not by adding more anchors within it.*

**Proof.**  A rotation about the line through collinear anchors fixes every anchor exactly (each anchor lies on the rotation axis and is therefore invariant), so it preserves every range $∥a_{k}-p_{tx}∥$ regardless of how many such collinear anchors are sampled, by the same argument as in Proposition 1 restricted to a single axis. Likewise, reflection through the plane containing coplanar anchors fixes every anchor in that plane exactly, so it preserves every range regardless of anchor count within the plane. □

Corollary 2 has direct practical relevance when the anchors are samples of a single moving platform, such as a calibration UAV, rather than several simultaneous distinct points: a straight-line transit or a constant-altitude flight (level flight, or a circular holding pattern) produces collinear or coplanar anchor samples respectively, however many samples are collected, and the corresponding ambiguity is never broken by collecting more of them. Genuine altitude or out-of-plane variation during the calibration pass—or a single additional stationary anchor outside the flight plane—is required to resolve the transmitter position fully. This is verified numerically in Section 4.8.

### 2.7. Image-Sharpness Objective

The linear delay-residual objective in (7) treats calibration as a parameter-estimation problem. For image formation, the quantity of interest is not parameter accuracy but the coherence of the matched-filter sum at each target location. Let

(10) $$Q\left(p;\;\left\{\delta q_{m}\right\},\left\{\delta t_{m}\right\}\right)=|a^{H}\left(p\right)\sum_{n=1}^{N}\bar{d}\left[n\right]\;x\left[n\right]{|}^{2}$$

denote the matched-filter score at candidate point p, evaluated using receiver positions and clock biases corrected by $\left\{\delta q_{m}\right\},\left\{\delta t_{m}\right\}$ (this is the same score used for image formation; see, e.g., [13] for the receiver-domain decomposition evaluation of Q(·) used elsewhere in this line of work). Define the coherent multistatic sharpness objective

(11) $$J\left(\left\{\delta q_{m}\right\},\left\{\delta t_{m}\right\},\left\{\delta p_{k}\right\}\right)=\sum_{k=1}^{K}Q\left(p_{k}^{0}+\delta p_{k};\;\left\{\delta q_{m}\right\},\left\{\delta t_{m}\right\}\right).$$

Maximizing *J* jointly over receiver and target corrections is a multistatic, multi-target generalization of classical phase-only autofocus, parameterized by physically meaningful geometric and timing corrections rather than by an unconstrained per-sample phase-error function. Two consequences follow directly from the identifiability analysis above and are worth stating explicitly. First, *J* is invariant under the same rotational null space as Proposition 1 (and, with only two anchors, under the residual axial rotation of Corollary 1): a rigid rotation of the whole constellation about $p_{tx}$ preserves every $\tau_{km}$ exactly, and hence preserves *J* exactly. Maximizing image sharpness alone therefore cannot resolve orientation any better than the linear delay model can; the anchors of Proposition 2 remain necessary. Second, *J* is phase-coherent and therefore periodic in each delay with period $1/f_{c}$, so it may have local maxima separated by range-cycle ambiguities that the envelope-based delay residual of Section 2.2 does not have. This motivates the two-stage algorithm of Section 3.

## 3. Algorithm

**Figure 2:**
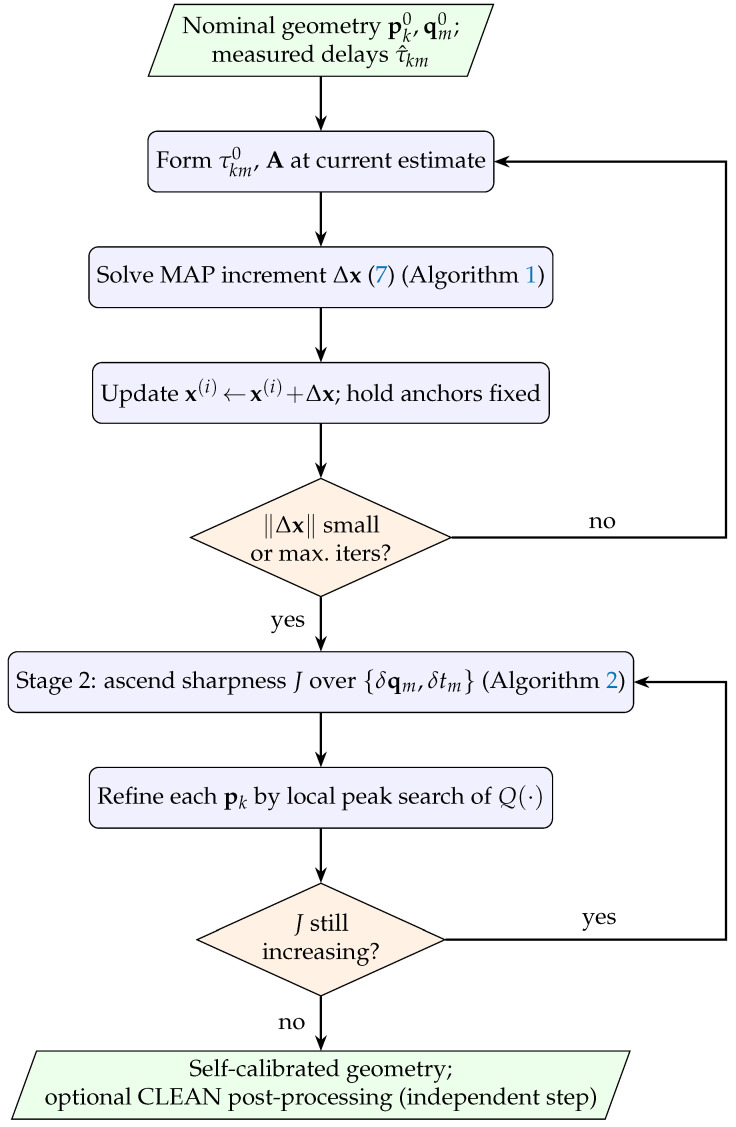
Flowchart of the two-stage self-calibration algorithm. Stage 1 (coarse, delay-residual MAP) and Stage 2 (fine, sharpness refinement) are each iterative loops; CLEAN, shown at the bottom, is an independent post-processing step applied afterward (Section 4.6), not part of the calibration loop itself.

The proposed procedure separates coarse, unambiguous correction from fine, phase-coherent refinement. Figure 2 summarizes the overall data flow, and Algorithms 1 and 2 give schematic pseudocode for each stage to make the structure below directly reproducible.

Stage 1 (coarse, delay-residual MAP). Using the envelope-based delay measurements ${\hat{\tau }}_{km}$ of (3), iterate the linearized MAP update of (7). Let $x^{\left(0\right)}=0$ denote the total accumulated correction relative to the original nominal geometry; at iteration *i*:

(i) Form $\tau_{km}^{0}$ and A at the current estimate (nominal geometry updated by $x^{\left(i\right)}$ so far).
(ii) Solve for the incremental correction $\Delta x={\left(A^{T}WA+\Sigma_{0}^{-1}\right)}^{-1}\left(A^{T}Wb-\Sigma_{0}^{-1}x^{\left(i\right)}\right)$.
(iii) Update the current estimate by Δx, and set $x^{\left(i+1\right)}=x^{\left(i\right)}+\Delta x$, holding the anchors of Proposition 2 fixed.
(iv) Repeat until ∥Δx∥ falls below a threshold or a maximum iteration count is reached.

**Algorithm 1** Stage 1: coarse delay-residual MAP correction**Require:** Nominal geometry $p_{k}^{0},q_{m}^{0}$; measured TOAs ${\hat{\tau }}_{km}$; noise weights W; prior covariance $\Sigma_{0}$; anchors fixed per Proposition 2; convergence threshold ϵ; max. iterations $I_{\max}$ **Ensure:** Converged correction $\hat{x}$  1: $x^{\left(0\right)}\leftarrow 0$  2: **for** $i=0,1,2,\ldots ,I_{\max}-1$ **do**  3:       Update nominal geometry by $x^{\left(i\right)}$; recompute $\tau_{km}^{0}$ and A at the new point   ▹ re-linearize  4:       $b\leftarrow \{c\;\delta {\hat{\tau }}_{km}\}$              ▹ range-equivalent residuals, see below  5:       $\Delta x\leftarrow {\left(A^{T}WA+\Sigma_{0}^{-1}\right)}^{-1}\left(A^{T}Wb-\Sigma_{0}^{-1}x^{\left(i\right)}\right)$ ▹ keep the $-\Sigma_{0}^{-1}x^{\left(i\right)}$ term—see caveat below  6:       $x^{\left(i+1\right)}\leftarrow x^{\left(i\right)}+\Delta x$, with anchor entries held at 0  7:       **if** ∥Δx∥<ϵ **then**  8:         **break**  9:       **end if** 10: **end for** 11: **return** $\hat{x}\leftarrow x^{\left(i+1\right)}$

The $-\Sigma_{0}^{-1}x^{\left(i\right)}$ term in step (ii) [line 5 of Algorithm 1] is essential and easy to omit by mistake: since $\Sigma_{0}$ is defined relative to the original nominal geometry, the prior must continue to penalize the full accumulated correction $x^{\left(i\right)}$ at every iteration, not merely each iteration’s local increment Δx. Omitting it does not cause the divergence that the sign or unit errors discussed below would; instead it silently removes the prior’s restoring effect after the first iteration, degrading accuracy and destroying the quadratic convergence rate expected of Gauss–Newton iteration. This is verified numerically in Section 4.2.

We also note, for implementers, that the clock-bias unknowns $\delta t_{m}$ should not be estimated directly in seconds alongside position corrections in meters: with typical priors (e.g., position errors of order $10^{-1}$–$10^{0}$ m and clock errors of order $10^{-9}$ s), the prior precisions $1/\sigma^{2}$ for the two unknown types differ by fifteen or more orders of magnitude, which is already at the edge of, or beyond, double-precision dynamic range and produces a catastrophically ill-conditioned $\left(A^{T}WA+\Sigma_{0}^{-1}\right)$. Reparameterizing the clock bias as a range-equivalent quantity $b_{m}=c\;\delta t_{m}$ (meters), and working entirely in range units throughout—including for the measurement residual, $c\;\delta {\hat{\tau }}_{km}$ rather than $\delta {\hat{\tau }}_{km}$ itself—removes this problem, since every unknown and every residual then shares a common physical scale; $b_{m}$ is converted back to a time bias, $\delta t_{m}=b_{m}/c$, only when reporting results.

Stage 2 (fine, sharpness refinement). Using Stage 1’s output as initialization, refine by alternating ascent on *J* of (11):

(i) With target position estimates fixed, update $\left\{\delta q_{m}\right\},\left\{\delta t_{m}\right\}$ by local ascent on *J* (e.g., gradient ascent or a short Gauss–Newton run on −J).
(ii) With receiver corrections fixed, refine each non-anchor $p_{k}$ independently by a local peak search of Q(·) around its current estimate.
(iii) Repeat until *J* stops increasing appreciably.

Because Stage 2 optimizes a periodic, non-convex objective, it is only reliable within a fraction of a wavelength of the true delay, which is why Stage 1 is required first: it removes the coarse geometric and timing error using an objective that has no such periodicity.

The receiver-domain decomposition method used elsewhere in this line of work for accelerated image formation is a natural candidate for evaluating Q(·) inside Stage 2’s inner loop, since *J* requires repeated evaluation of the matched-filter score at trial geometries. This is not free, however: the phase reference used by that decomposition depends on receiver-subaperture geometry, and must be recomputed whenever $\left\{\delta q_{m}\right\}$ changes, which couples the two methods’ computational costs in a way that is not yet quantified and is left for future work.
**Algorithm 2** Stage 2: fine phase-coherent sharpness refinement**Require:** Stage 1 output $\hat{x}$ (initial $\left\{\delta q_{m},\delta t_{m},\delta p_{k}\right\}$); sharpness objective J(·) of (11); convergence tolerance η **Ensure:** Refined $\left\{\delta q_{m},\delta t_{m},\delta p_{k}\right\}$  1: Initialize $\left\{\delta q_{m},\delta t_{m},\delta p_{k}\right\}$ from $\hat{x}$  2: $J_{\mathrm{prev}}\leftarrow J\left(\left\{\delta q_{m}\right\},\left\{\delta t_{m}\right\},\left\{\delta p_{k}\right\}\right)$  3: **repeat**  4:    With $\left\{\delta p_{k}\right\}$ fixed, ascend *J* over $\left\{\delta q_{m},\delta t_{m}\right\}$ (gradient ascent or short Gauss–Newton on −J)     ▹ Stage 2, step (i); sensitive to initialization—see Section 3.1  5:    **for** each non-anchor target *k* **do**  6:         Refine $\delta p_{k}$ by a local peak search of $Q(\cdot ;\left\{\delta q_{m}\right\},\left\{\delta t_{m}\right\})$ around the current estimate      ▹ Stage 2, step (ii)  7:    **end for**  8:    $J_{\mathrm{cur}}\leftarrow J\left(\left\{\delta q_{m}\right\},\left\{\delta t_{m}\right\},\left\{\delta p_{k}\right\}\right)$  9:    $\Delta J\leftarrow J_{\mathrm{cur}}-J_{\mathrm{prev}}$; $J_{\mathrm{prev}}\leftarrow J_{\mathrm{cur}}$ 10: **until** ΔJ<η 11: **return** $\left\{\delta q_{m},\delta t_{m},\delta p_{k}\right\}$

### 3.1. Computational Complexity and Sensitivity to Initialization

#### 3.1.1. Stage 1

Each Gauss–Newton iteration (Algorithm 1) forms A, an (MK)×(3K+4M) sparse matrix with exactly two nonzero position-gradient blocks and two nonzero clock-bias entries per row (Section 2.2), so assembling A and the normal-equations matrix $A^{T}WA$ costs O(MK). Because different target/receiver blocks of x interact only through shared rows, $A^{T}WA$ is itself sparse (block structure induced by the target–receiver bipartite observation pattern) rather than dense, but the direct solve of the (3K+4M)×(3K+4M) system ${\left(A^{T}WA+\Sigma_{0}^{-1}\right)}^{-1}$ in step (ii) is, in the worst case (a dense Cholesky factorization ignoring this structure), $O\left({\left(K+M\right)}^{3}\right)$ per iteration; exploiting the sparsity pattern with a sparse Cholesky or conjugate-gradient solve reduces this substantially in practice for the receiver counts used in Section 4 (M=40, K=10, giving a 190×190 system), though we report the conservative dense-solve bound here since we have not benchmarked a sparse implementation specifically. The number of Gauss–Newton iterations to convergence is small and essentially independent of problem size once the recentering term of step (ii) is included (six iterations to machine precision in Section 4.2), so Stage 1’s total cost is dominated by a small constant number of $O\left({\left(K+M\right)}^{3}\right)$ solves, not by an unbounded iteration count.

#### 3.1.2. Stage 2

Each ascent step on *J* (Algorithm 2, step (i)) requires evaluating Q(·), and hence a coherent sum over all *M* receivers, at each of the *K* target locations for every trial value of $\left\{\delta q_{m},\delta t_{m}\right\}$ probed by the local optimizer (e.g., one evaluation per gradient-ascent step, or several per Gauss–Newton line search), giving a per-evaluation cost of O(MK) and a total Stage 2 cost of $O(MK\cdot N_{\mathrm{eval}})$, where $N_{\mathrm{eval}}$ is the number of trial evaluations to convergence—itself problem- and implementation-dependent, since *J* is non-convex (Section 2.7). Step (ii)’s per-target local peak search of Q(·) around the current estimate adds $O(M\cdot N_{\mathrm{grid}})$ per target, where $N_{\mathrm{grid}}$ is the number of candidate points probed in the local search box; this is the same per-point cost as ordinary matched-filter image formation, restricted to a small neighborhood rather than the full imaging volume.

#### 3.1.3. Sensitivity of Stage 2 to Initialization and Local Optima

Unlike Stage 1, whose delay-residual objective is quadratic (hence has a single, global optimum for a fixed linearization point), Stage 2’s sharpness objective *J* is periodic in each delay with period $1/f_{c}$ (Section 2.7) and therefore has local maxima spaced roughly one range-cycle apart ($c/f_{c}$ in range, i.e., the carrier wavelength). Stage 2 is reliable only when its starting point—Stage 1’s output—is already within a fraction of a wavelength of the true correction, which is precisely what Stage 1’s carrier-independent delay-residual solve is designed to guarantee (Section 4.2 shows Stage 1 alone converging to sub-wavelength position accuracy). If Stage 2 were instead initialized from a coarser estimate—for instance, skipping Stage 1 entirely, or initializing from a nominal geometry whose error is a significant fraction of $c/f_{c}$ or larger—it could converge to the wrong range-cycle, a local rather than global maximum of *J*, with no local diagnostic (gradient norm, Hessian conditioning) distinguishing this from correct convergence, in the same spirit as the mirror-twin ambiguity of Section 4.8 being invisible to a rank check. We have not characterized this failure mode quantitatively (e.g., the fraction of random coarse initializations that converge to the wrong range-cycle as a function of initial error), since every experiment in Section 4 initializes Stage 2 from converged Stage 1 output as the algorithm is designed to be used; doing so is a natural extension for future work.

## 4. Numerical Results

This section reports numerical experiments verifying the identifiability analysis of Section 2.5 and the convergence behavior of Stage 1 (Section 3), and demonstrating the effect of self-calibration, bandwidth/windowing, and CLEAN deconvolution on a simulated multistatic image of an extended target. All experiments use $c=3\times 10^{8}$ m/s and a representative near-field multistatic geometry: a transmitter at the origin and M=40 receivers on a ring of radius 400 m, the transmitter and receivers both in the z=0 plane, together with K=10 calibration targets at nominal ranges of a [100,400] m. Section 4.1 and Section 4.2 verify Stage 1 (Section 3) specifically, which is carrier-frequency-independent by deliberate construction—it works directly in the delay/range domain precisely so that it has no periodic ambiguity, which is what lets it supply Stage 2 with a starting point already accurate to within a fraction of a wavelength (Section 3); this says nothing about Stage 2 itself, which does depend on carrier frequency/wavelength through *J* and Q(·). Section 4.4, Section 4.5 and Section 4.6 simulate the coherent image itself, which requires a carrier frequency (here 4 GHz), while the real target being imaged (the eagle silhouette’s scatterers) is elevated to a representative 500 m flying altitude above Earth.

### 4.1. Identifiability Verification

**Figure 3:**
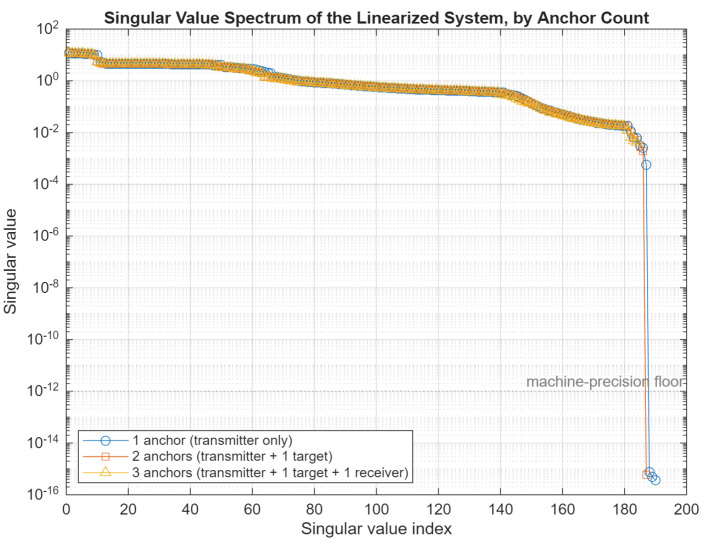
Singular value spectrum of the linearized system A with one, two, and three anchors. The count of near-machine-precision singular values (3, 1, 0) matches Proposition 1, Corollary 1, and Proposition 2 exactly.

Figure 3 shows the singular value spectrum of the linearized system matrix A from (6), computed for the same nominal geometry with (a) one anchor (transmitter only), (b) two anchors (transmitter plus one target), and (c) three anchors (transmitter, one target, one receiver, non-collinear). The results confirm Section 2.5 exactly: with one anchor, the three smallest singular values are at machine precision (∼$10^{-16}$ relative to the largest), matching the three-dimensional null space of Proposition 1; with two anchors, exactly one singular value remains at machine precision, matching the one-parameter axial rotation of Corollary 1; and with three anchors, no singular value is near zero, and the condition number of A over the entire spectrum is a modest 2879, matching Proposition 2. This is a purely structural property of the geometry and receiver–target association—it is computed here from the noiseless Jacobian alone, before any measurement is simulated.

### 4.2. Stage 1 Convergence

**Figure 4:**
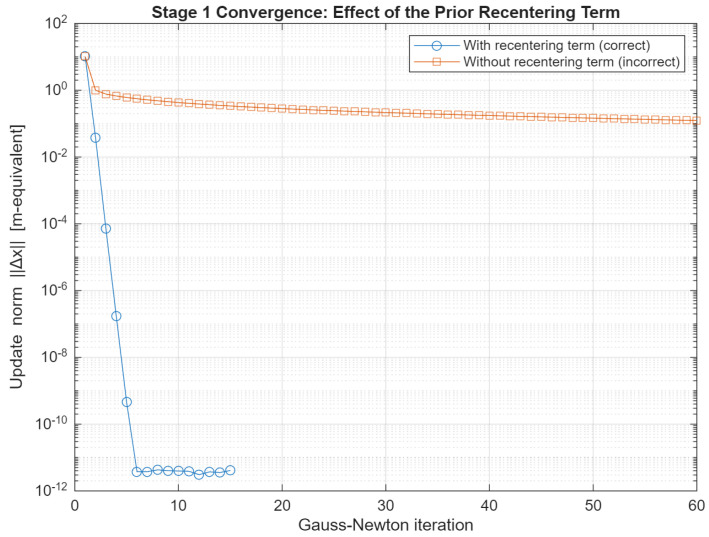
Stage 1 Gauss–Newton update norm ∥Δx∥ (range-equivalent units, log scale) versus iteration, with and without the $-\Sigma_{0}^{-1}x^{\left(i\right)}$ recentering term. With the term, convergence is quadratic to machine precision in six iterations; without it, convergence is slow, non-monotonic in the individual parameter blocks, and does not complete after 60 iterations.

Figure 4 illustrates the practical importance of the $-\Sigma_{0}^{-1}x^{\left(i\right)}$ recentering term in the Stage 1 update (Section 3). With the term included, Gauss–Newton iteration converges quadratically: starting from position and clock-bias errors with RMS values of 0.66 m (calibration targets), 0.74 m (receivers), and 5.3 ns (clocks), the update norm decreases from 10.4 m to machine precision ($4\times 10^{-12}$ m) in six iterations, and the final RMS errors are 0.07 m, 0.50 m, and 1.5 ns respectively. Without the recentering term—i.e., solving $\Delta x={\left(A^{T}WA+\Sigma_{0}^{-1}\right)}^{-1}A^{T}Wb$ at each iteration, which looks like a natural implementation of (7) if the accumulation issue is overlooked—the update norm decreases only slowly and non-monotonically and does not converge after 60 iterations (update norm still 0.054 m), and, notably, the receiver-position RMS error increases over the course of the iterations (from 0.74 m to 1.54 m) even as the target-position and clock-bias errors continue to improve. This asymmetric failure mode—some parameter blocks improving while others silently degrade—is a practical hazard worth checking for explicitly in any implementation of (7) as an iterative loop rather than a single linear solve.

### 4.3. CRLB Comparison

To quantify how close the two-stage estimator of Section 3 comes to the best achievable accuracy, we compare its empirical root-mean-square (RMS) error, over 500 independent Monte Carlo trials per condition, against the Bayesian CRLB of (9), evaluated at the anchored, post-convergence geometry (the three anchors of Proposition 2 fixed), using the same nominal geometry and prior covariance $\Sigma_{0}$ as Section 4.2. Rather than a single operating point, we sweep two quantities that determine the achievable accuracy directly: the per-measurement time-of-arrival SNR, and the number of calibration-target samples *K*. With 500 independent trials per condition, the relative standard error of the empirical RMS estimate itself is approximately $1/\sqrt{2500}$ (the standard result for the sampling variability of an RMS/standard-deviation estimate under approximately Gaussian errors), which we report alongside the point estimates below rather than leaving the trial count and its associated uncertainty implicit.

**Figure 5:**
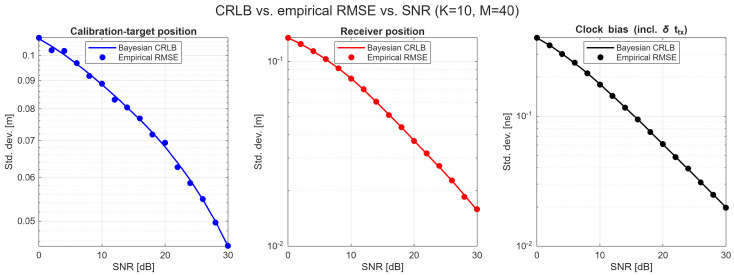
Per-block Bayesian CRLB standard deviation (lines) versus empirical RMS error (markers), as a function of time-of-arrival SNR, for calibration-target position, receiver-position, and clock-bias (including $\delta t_{tx}$) blocks, at fixed *K* and *M*. Each panel carries its own legend identifying the parameter block and the CRLB-vs.-empirical distinction.

**Figure 6:**
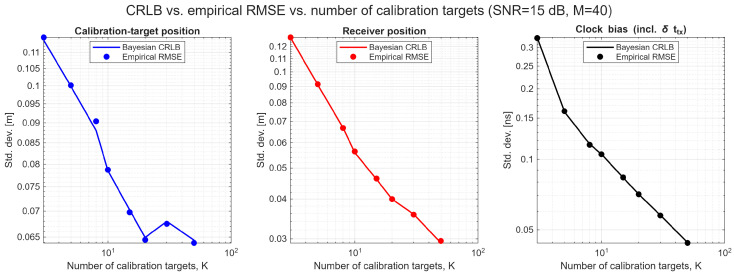
Per-block Bayesian CRLB standard deviation (lines) versus empirical RMS error (markers), as a function of the number of calibration-target samples *K*, at a fixed operating SNR. Each panel carries its own legend identifying the parameter block and the CRLB-vs.-empirical distinction.

Figure 5 plots the per-parameter-block CRLB standard deviation, $\sqrt{{\left[J_{\mathrm{MAP}}^{-1}\right]}_{ii}}$ (averaged within each of the calibration-target position, receiver-position, and clock-bias blocks, the latter including the transmit-time offset $\delta t_{tx}$ introduced in Section 2), against SNR, holding *K* and *M* fixed at their values from Section 4.2, alongside the empirical RMS error at each SNR. As expected, all three curves fall as $1/\sqrt{\mathrm{SNR}}$ at a high SNR, flattening toward the prior-dominated floor set by $\Sigma_{0}$ at a low SNR. Figure 6 plots the same quantities against the number of calibration-target samples *K* at a fixed operating SNR, showing the expected $1/\sqrt{K}$-type improvement from measurement redundancy once enough anchors are present to keep $J_{\mathrm{MAP}}$ well conditioned.

In both sweeps, the empirical RMS error tracks the Bayesian CRLB to within 2.6% across the full range tested (median deviation 0.5%; SNR-sweep and *K*-sweep maxima, 2.1% and 2.6% respectively, agree closely enough to report a single combined figure), with a $\pm 1/\sqrt{2500}$ relative sampling uncertainty on each empirical point as noted above, consistent with the remark following (9): since (7) is the linear estimator that attains (9) with equality for a linear Gaussian model, the residual gap is attributable to the re-linearization error of Gauss–Newton iteration on the nonlinear model (2) and (3) rather than to any inefficiency of the estimator itself.

### 4.4. Effect of Self-Calibration and Sidelobe Suppression on a Simulated Extended-Target Image

To illustrate the effect of self-calibration on image quality rather than on parameter accuracy alone, we simulate a multistatic matched-filter image of an extended target: a synthetic eagle silhouette (wings spread, in a soaring/banking pose) represented as 900 point scatterers, elevated to a representative 500 m flying altitude above a ground-based (z=0) transmitter and M=40 receivers on a 400 m radius ring, imaged with a 4 GHz carrier. A handful of these scatterers—the head, tail, both wingtips, and the body center, identified automatically as the extremal points along the target’s principal axes—are given six times the amplitude of the remaining, roughly uniform background scatterers, so that the simulated target has a small number of dominant reflectors rather than 900 roughly equal-amplitude points; this is a closer match to how real extended targets (aircraft, ships) typically image, with a handful of strong corner-reflector-like returns standing out against a weaker diffuse background, and it is what CLEAN (Section 4.6) is intended to exploit. Receiver positions carry a 20 cm (per-axis) position error and a 0.5 ns clock-bias error, both comparable to or larger than the carrier wavelength (7.5 cm) and therefore expected to defocus the image substantially if uncorrected. One receiver position is treated as an exact anchor, consistent with Section 2.5. Results are displayed over a 4×4 m field of view so that the ∼1.2 m target is seen against a background rather than filling the frame.

**Figure 7:**
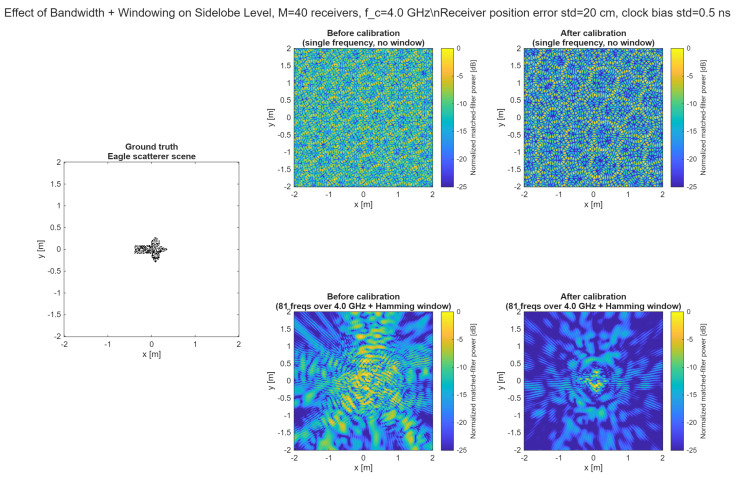
Ground truth (eagle silhouette, (**left**)) and matched-filter images before/after self-calibration, comparing a single carrier frequency with no windowing (**top row**) against 81 stepped-frequency samples over a 4 GHz bandwidth with Hamming windowing in both the aperture and frequency domains (**bottom row**). Colorbars show normalized matched-filter power in dB.

With only self-calibration applied (Figure 7, top row), the coherent image-sharpness objective *J* of (11), evaluated at the true scatterer locations, improves by a factor of 1.12× from before to after correction, confirming that the calibration improves not just parameter accuracy (Section 4.2) but the coherence of the resulting image, which is the quantity of practical interest for imaging. However, with only M=40 receivers at a single carrier frequency, the resulting image (both before and after correction) is dominated by the sparse ring aperture’s own diffraction (grating-lobe-like) sidelobe pattern, which is visually difficult to distinguish from the target even after correction. This is expected: a single-frequency, unwindowed sparse aperture has an intrinsically high sidelobe floor, independent of how accurately the receiver geometry is known.

### 4.5. Effect of Bandwidth and Windowing on Sidelobe Level

We evaluate two standard remedies for the sidelobe floor identified above, applied together: (i) bandwidth, replacing the single carrier with $N_{f}=81$ stepped-frequency samples spanning B=4 GHz, which is equivalent to coherent range-profile synthesis (pulse compression) and decorrelates the single-tone sidelobe pattern, and (ii) windowing, a Hamming taper applied across both the *M* receivers (aperture domain) and the $N_{f}$ frequencies (frequency domain), the standard radar tradeoff of a modestly wider mainlobe for substantially lower sidelobes.

Table 2 and Figure 7 summarize the effect, evaluated after self-calibration in all cases. Both the peak-to-mean ratio and the correlation of the image with the true scatterer scene improve substantially with bandwidth and windowing; the sharpness improvement contributed by self-calibration itself also increases, from 1.12× to 2.99×, once the sidelobe floor is no longer masking it.

### 4.6. CLEAN Deconvolution

We clarify at the outset how this subsection’s results should be read: CLEAN is applied here as an independent, post-processing image-domain enhancement, not as an integrated component of the self-calibration estimator of Section 2 and Section 3. Every correction to receiver position, clock bias, and target position is already fixed by that estimator before CLEAN ever runs; CLEAN then deconvolves the resulting (already self-calibrated) image and has no influence on, and receives no information back from, the calibration parameters themselves. Any improvement attributed to CLEAN below is therefore strictly additional to, and separable from, the self-calibration gains reported in Section 4.4 and Section 4.5, which is also why Table 3 reports it against the self-calibrated dirty image rather than against the uncalibrated one.

Bandwidth and windowing suppress sidelobes by construction (i.e., by changing how the dirty image is formed), but do not remove the residual point-spread-function structure of the finite, sparse aperture. We additionally apply Hogbom CLEAN [12]—to our knowledge, not previously combined with geometry/timing self-calibration of the kind developed in this paper—as an image-domain deconvolution step on top of the wideband, windowed, self-calibrated image of Section 4.5.

Two implementation points proved essential and are reported here since both failure modes could easily recur in a different implementation. First, CLEAN’s peak-find-and-subtract step assumes the dirty image is a linear convolution of the true scene with the point-spread function (PSF); this linearity holds only in the complex coherent-sum domain, not after the modulus-squared (power/intensity) operation used for display, so CLEAN must operate on the complex image, with power computed only at the end. Second, an unrestricted peak search across the full field of view is numerically unstable for an extended, non-uniformly reflective target of this kind: the residual peak, which should decrease monotonically, instead grew substantially over the iteration when the search was unrestricted, because the search wandered into background regions whose sidelobe structure does not match the space-invariant PSF assumed for subtraction. Restricting the peak search to a box of radius 0.7 m around the known approximate target location—standard practice in radio-interferometric imaging—restores monotonic convergence, reaching it in 1253 iterations.

**Figure 8:**
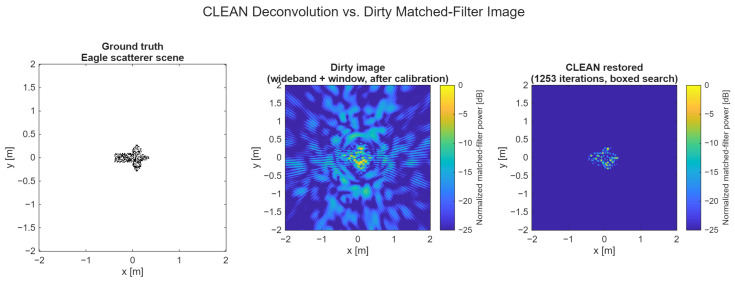
Ground truth (eagle silhouette, (**left**)), dirty image (wideband, windowed, self-calibrated; (**center**)), and CLEAN-restored image (boxed peak search; (**right**)). Colorbars show normalized matched-filter power in dB.

Table 3 and Figure 8 compare the wideband, windowed, self-calibrated dirty image against the CLEAN-restored image. The peak-to-mean ratio improves by more than an order of magnitude (103.1→1665.8), consistent with CLEAN concentrating energy onto a small number of discrete restored components (the same handful of dominant scatterers introduced in Section 4.4). Correlation with the true scene, by contrast, is slightly lower after CLEAN (0.348→0.291): the correlation reference is a binary mask of all 900 scatterer locations, most of which are the roughly uniform, non-dominant background rather than the five boosted landmarks, so an image that increasingly concentrates its energy onto those five points—exactly what CLEAN is designed to do—necessarily resembles that full, diffuse 900-point mask somewhat less, even as it more accurately reconstructs the target’s actual strongest reflectors. This is the same limitation noted qualitatively above: the target’s own large internal reflectivity variation (a handful of dominant scatterers against a much weaker diffuse background) limits how closely any single per-pixel amplitude measure, evaluated against the full scatterer footprint, can track CLEAN’s increasingly sparse reconstruction. Entropy, unlike correlation, agrees with the peak-to-mean story: it drops substantially after CLEAN (10.71→6.20), consistent with CLEAN redistributing the image’s energy from a broad, high-entropy sidelobe-and-background spread onto a small, low-entropy set of restored components.

### 4.7. Target Resolvability: Two Instances of the Eagle Target

The two fixes described in an earlier revision (matching against each target’s nearest dominant-scatterer landmark rather than its overall centroid; a pixel-count floor on the peak-detection non-max-suppression radius) were confirmed by re-running the corrected script: the results below are from that corrected run.

Section 4.4, Section 4.5 and Section 4.6 concern a single extended target; a separate and practically important question is whether the same self-calibrated multistatic system can resolve multiple, simultaneous, discrete targets rather than merging them into one blob. We evaluate this with a second scene: $K_{b}=2$ copies of the same eagle target used above, placed 4 m apart at (∓2,0) m (1800 scatterers total, each copy carrying the same dominant-scatterer landmarks as Section 4.4), observed by the same M=40-receiver geometry, receiver-position/clock errors, and self-calibration procedure, and processed identically through bandwidth/windowing (Section 4.5) and a single CLEAN pass (Section 4.6) applied after self-calibration, with the peak search restricted to the union of two small boxes (one per target, the same box size validated for the single eagle) rather than one box spanning the full separation.

**Figure 9:**
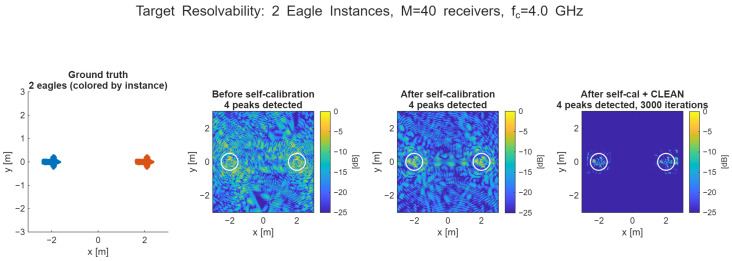
Ground truth 2-eagle scene, 4 m apart (left, each of the two target instances rendered in a distinct color, red and blue; true centroids marked in the remaining panels by an open circle of 0.5 m radius, drawn in the same color as its corresponding instance so the two circles can be matched to the ground-truth panel at a glance), and matched-filter images before self-calibration, after self-calibration, and after self-calibration plus CLEAN (remaining three panels): both instances are resolved as separate peaks once self-calibration is applied.

The relevant question is not image sharpness at a single target’s scatterers (as in Section 4.4) but whether each of the two eagle instances is recovered as a separate local maximum of the image, at approximately its true centroid, rather than the two instances merging into one blob or a single instance splitting into spurious multiple peaks. We report: (i) the number of detected local maxima above a given threshold versus the true count (2); (ii) the mean and worst-case position error between each detected peak and its nearest true centroid; (iii) whether the 4 m separation is resolved as two distinct peaks, compared against the Rayleigh-type resolution limit set by the 400 m ring aperture and carrier wavelength at 500 m range; and (iv) how each of these changes before self-calibration, after self-calibration, and after self-calibration followed by CLEAN, since uncorrected receiver-position/clock errors are exactly the kind of defocusing that would be expected to merge closely spaced targets that would otherwise be resolved, and CLEAN is applied here specifically to test whether it improves resolvability further, not only single-target sharpness (see Figure 9 and Table 4).

Before self-calibration, neither target is matched within one mainlobe width (0.045 m at this bandwidth): the uncorrected receiver-position and clock errors defocus the image enough that the 4 m separation is not recovered at all (an effectively infinite/undefined minimum resolved separation). After self-calibration, both targets are correctly matched, mean position error drops by roughly 7× (from 0.229 m to 0.031 m), and the true 4 m separation is recovered exactly. CLEAN applied on top of self-calibration gives essentially no further improvement in this resolvability sense—mean error is marginally better (0.028 m) but worst-case error is marginally worse (0.038 m)—consistent with CLEAN’s benefit (Section 4.6) being concentrated in single-target image sharpness and sidelobe suppression rather than in multi-target position accuracy, which self-calibration alone already resolves at this separation. One caveat on the peak-detection count itself is that four peaks are detected against only two true targets in every condition, including after correction; since each eagle carries five close-together dominant scatterers rather than one, more than one of them can individually clear the peak threshold within a single target’s own footprint, so the raw detected-peak count over-counts true targets here by design of the target model, not as a resolvability failure—the more informative figure is the number of true targets matched within one mainlobe (two of two after correction), not the raw peak count.

### 4.8. Transmitter Localization from Known Anchors

Section 2.6 predicted that localizing an unknown transmitter from a set of exactly known anchors depends on both the anchors’ geometric spread and whether the transmitter’s emission time $t_{0}$ is known, with a genuine ambiguity (not merely poor conditioning) when the anchors are collinear or coplanar. We verify this for the practically relevant case of a single moving anchor—e.g., a calibration UAV—sampled at many time instants, comparing three flight geometries against a fixed true transmitter position, using the same rank and mirror-reflection tests introduced in Section 4.1.

For a straight-line transit (50 collinear samples), the linearized system retains rank 2 regardless of sample count, with the third singular value at machine precision (<$10^{-12}$ relative to the largest): the predicted one-parameter axial ambiguity of Corollary 2 is exact, not merely ill-conditioned. For constant-altitude flight (50 coplanar samples on a circular orbit), the linearized system reaches full rank 3—which would appear, from the rank test alone, to indicate a well-posed problem—but an explicit reflection of the true transmitter position through the sampled flight-altitude plane reproduces every one of the 50 range measurements exactly, to floating-point precision (maximum residual exactly 0.0 in double precision): this is an algebraic identity, not a numerical coincidence—a discrete twin solution that a rank or conditioning check alone would miss entirely, since it is a global rather than local phenomenon. Only genuine three-dimensional maneuvering (12 samples with substantial altitude variation) breaks the reflection ambiguity outright, with the same mirror construction now reproducing the ranges only to within 9.3 m—clearly distinguishable from the true solution given realistic measurement noise.

This result has a direct practical implication for calibration campaigns using a single moving reference rather than several simultaneous distinct anchors: level flight and circular holding patterns, arguably the most common default UAV flight behaviors, are exactly the coplanar degenerate case, and the resulting ambiguity will not be revealed by monitoring the linearized system’s rank or condition number, since both appear nominal. A calibration flight plan should therefore include deliberate, sufficient altitude or out-of-plane variation, or incorporate at least one stationary anchor outside the flight plane, rather than relying on sample count alone.

## 5. Discussion

The results in Section 4 separate three distinct sources of image degradation that are easy to conflate in practice, and show that each requires a different remedy. Geometric and timing errors (receiver position and clock bias) are addressed by the self-calibration framework of Section 2 and Section 3, and their effect is directly visible in the sharpness improvement of Section 4.4. Sparse-aperture, finite-bandwidth sidelobe structure is a separate effect, present even with perfect geometric knowledge, and is addressed by bandwidth and windowing (Section 4.5), not by any amount of further geometric refinement—Table 2 shows the self-calibration gain itself increasing once the sidelobe floor is suppressed, indicating that the two effects had previously been partially masking one another. Finally, the residual point-spread structure of the (now well-suppressed but not eliminated) aperture is addressed by CLEAN deconvolution (Section 4.6), which is a further, distinct correction rather than a substitute for either of the first two. A practical implication is that comparing self-calibration methods, or judging whether a calibration algorithm is “working,” on final image appearance alone can be misleading unless the bandwidth, windowing, and deconvolution stages are held fixed across the comparison; Section 4.4, Section 4.5 and Section 4.6 report each contribution in isolation for this reason.

The identifiability results of Section 4.1 are, by construction, properties of the noiseless linearized geometry and are independent of the imaging experiments of Section 4.4, Section 4.5 and Section 4.6; the two are connected only through Section 2.7’s observation that the image-sharpness objective inherits the same rotational null space as the delay-residual objective. This separation is deliberate: it means the anchor requirements of Propositions 1 and 2 can be checked once, analytically or via the singular value diagnostic of Figure 3, for a given sensor deployment, independent of the specific scene or waveform used for any particular imaging run.

The convergence failure mode documented in Section 4.2—slow, asymmetric degradation of one parameter block while others continue to improve, rather than an obvious catastrophic divergence—is arguably more dangerous in practice than the unit-scaling and sign errors also encountered in developing this framework (and corrected in Section 3), precisely because it does not announce itself: an implementation with this bug can appear to be converging (the target-position and clock-bias errors do improve) while silently accumulating error in the receiver-position estimates. We recommend that any implementation of the Stage 1 iteration log the per-block (target, receiver, clock) update norms separately, not only the aggregate ∥Δx∥, to catch this failure mode early.

The CLEAN deconvolution results should be read with the same caveat given in Section 4.6: the search-box restriction that makes the method numerically stable requires an approximate target location as input, which in a real system would come from a coarse detection or region-of-interest stage rather than from prior knowledge of the answer. This is a legitimate two-stage design—coarse detection followed by refinement, analogous to Stage 1 followed by Stage 2 in the self-calibration algorithm itself—but it is a real dependency of the method, not a limitation that CLEAN removes.

Finally, the two-stage self-calibration algorithm and the CLEAN deconvolution step are, at present, applied sequentially rather than jointly: the imaging experiments of Section 4.4, Section 4.5 and Section 4.6 use self-calibration to correct geometry and timing, and separately apply bandwidth, windowing, and CLEAN to the resulting image. Whether the self-calibration objective itself would benefit from being defined on a CLEAN-deconvolved image rather than on the raw matched-filter score Q(·)—potentially improving the conditioning of Stage 2’s sharpness refinement, at the cost of coupling the two methods’ computational loops—is an open question left for future work.

The transmitter-localization results of Section 4.8 carry a practical warning that is easy to overlook precisely because it does not show up in the usual diagnostic: a rank or condition-number check on the linearized system, of the kind recommended throughout this paper for the main self-calibration problem, is a purely local test and cannot detect the coplanar reflection ambiguity of Corollary 2, since both the true transmitter position and its mirror twin are individually well-conditioned, isolated solutions—the ambiguity is global, between two distinct points, not a local flattening of the objective around either one. A calibration campaign relying on rank-checking alone to certify that “enough” anchor samples have been collected could therefore converge confidently to the wrong, reflected transmitter position without any indication in the numerics that anything is wrong. This reinforces a theme already present in Section 4.2: the absence of a warning sign in a diagnostic is not evidence of correctness unless the diagnostic is known to be sensitive to the specific failure mode in question.

### From Synthetic Measurements to Real Ones

Every numerical result in Section 4 uses synthetic delay measurements: additive Gaussian noise on an otherwise exact bistatic delay (2), with time of arrival extracted (implicitly) as a noiseless matched-filter peak plus noise. Real time-of-arrival estimation from matched filtering departs from this idealization in at least four ways that the present framework does not yet model explicitly. First, multipath introduces additional, spurious correlation peaks displaced from the direct-path delay by a propagation-environment-dependent amount; if a multipath peak is mistaken for the direct-path echo, the resulting ${\hat{\tau }}_{km}$ carries a systematic, nonzero-mean bias rather than the zero-mean noise assumed in (3), which is precisely the outlier scenario the IRLS-style remedy sketched in Section 2.2 is intended to mitigate, though we have not evaluated multipath-specific delay statistics numerically here. Second, clutter (unwanted returns from the background scene rather than the intended calibration target) can produce a competing correlation peak of comparable or greater amplitude than the true target echo, which is a target-association error (attributing a clutter delay to calibration target *k*) rather than a delay-estimation error per se; the present framework assumes correct target–receiver association is already resolved upstream of the delay-residual model, and does not include a robust association/data-association step. Third, missed detections (a calibration target’s echo falling below the detection threshold at some receiver, e.g., due to an unfavorable bistatic geometry or fading) simply remove the corresponding row of (6) from the observation set; because the identifiability analysis of Section 2.5 already treats the anchor/receiver–target observation pattern generally, a missed detection is handled correctly by the existing framework provided enough of the remaining pairs still satisfy Propositions 1 and 2, but a real system should verify this by recomputing the realized singular value spectrum on the actual available measurement set at run time, not merely assume it from the nominal (fully connected) receiver–target graph used in Section 4. Fourth, inaccurate delay-peak extraction—from finite sampling-rate quantization, imperfect interpolation of the matched-filter output near its peak, or a low-SNR peak location bias distinct from ordinary additive noise—degrades the effective per-pair measurement precision represented by W in (6) and (7); because the estimator already accepts an arbitrary, possibly heterogeneous W, this can be absorbed by using a realistic, possibly receiver-pair-dependent noise covariance rather than a single scalar SNR, but doing so requires correctly characterizing the true delay-estimation error statistics of the specific waveform and receiver hardware in use, which is outside the scope of the synthetic study reported here. Taken together, these four effects are the principal reason we present the numerical results of Section 4 as a controlled verification of the theoretical predictions of Section 2 and Section 3 rather than as a claim of validated real-world performance; validating the framework against measured or experimentally realistic data—ideally including deliberately introduced multipath, clutter, and missed detections—is an important direction for future work.

## 6. Conclusions

This paper formulated joint self-calibration of receiver position, receiver clock bias, and target position for multistatic radar as a maximum a posteriori estimation problem driven by bistatic delay residuals, with measurement noise and prior parameter uncertainty represented separately. We characterized the gauge freedom of this problem progressively: with only the transmitter known exactly, an exact three-dimensional rotational null space; with one additional anchor, a one-parameter axial rotation ambiguity; and with two additional anchors (one target, one receiver, non-collinear with the transmitter), the full removal of continuous ambiguity. We also treated the dual problem of localizing an unknown transmitter from a small number of exactly known anchors—receivers, targets, or time samples of a single moving platform—showing that this reduces to classical multilateration rather than to a continuous gauge freedom, but that collinear or coplanar anchor geometries leave an uncorrectable continuous or discrete ambiguity, respectively, regardless of anchor count, and that the discrete (coplanar) case is invisible to a standard rank or conditioning check. We reformulated the calibration objective in terms of coherent multistatic image sharpness rather than parameter accuracy, connecting it directly to the matched-filter score used for image formation, and proposed a two-stage coarse-then-coherent estimation algorithm.

Numerical experiments verified each of these claims directly: the singular value spectrum of the linearized system showed exactly three, one, and zero near-machine-precision singular values at one, two, and three anchors respectively; the proposed Gauss–Newton iteration converged quadratically to machine precision, while an implementation omitting the prior-recentering term converged slowly and let the receiver-position error silently increase; a simulated multistatic image of an extended (eagle-shaped) target showed measurable sharpness improvement from self-calibration alone, with substantially better image quality once bandwidth, aperture/frequency windowing, and CLEAN deconvolution were applied on top of the corrected geometry; a second scene with multiple simultaneous discrete targets (two instances of the same eagle target) tested whether self-calibration improves not just single-target focus but the resolvability of closely-spaced distinct targets; and the transmitter-localization experiments showed an exact (not approximate) mirror-twin solution for coplanar anchor samples such as level UAV flight, resolved only by genuine three-dimensional anchor spread.

The present treatment has several limitations that bound its current scope. The analysis assumes small errors (first-order linearization) and Gaussian noise and priors; it does not address large initial errors, non-Gaussian outliers in target–receiver association, or moving targets. Section 2.2 gives a qualitative characterization of how large an initial error the linearization tolerates (governed by the correction magnitude relative to the transmitter–target/target–receiver range), but we have not mapped the Gauss–Newton basin of convergence numerically; large initial errors that violate this regime would require a coarser, nonlinearized search or a damped/trust-region variant before (7) applies directly, as discussed there. The discrete reflection ambiguity noted in Proposition 2 is argued to be locally benign but is not algorithmically enforced. The CLEAN deconvolution step requires an approximate target region as input and has not been jointly optimized with the self-calibration objective itself. The numerical validation in Section 4 uses a single representative geometry (M=40 receivers on a ring, K=10 calibration targets); while the identifiability analysis of Section 2.5 and the algorithm of Section 3 are derived for a general receiver–target configuration and are not specific to this geometry, we have not numerically verified their behavior on the sparse, irregular, or non-uniform deployments typical of many real multistatic radar installations, and generalizing the numerical study to such geometries—particularly cases closer to the degenerate anchor configurations of Corollary 2—is left for future work. Similarly, the calibration targets used throughout are treated as a small, dedicated set of stationary reference points; the framework as given does not yet address using a large number of arbitrary, opportunistically available scene scatterers directly as calibration references (as opposed to a coarse image estimate of a few dedicated targets, discussed in Section 2.2), which would require an association/outlier-rejection layer beyond the scope of the present paper, since arbitrary scene scatterers are not guaranteed to be individually resolvable or correctly associated across receivers the way dedicated calibration targets are assumed to be here. Finally, Section 5 discusses several specific real-measurement effects—multipath, clutter, missed detections, and delay-peak extraction error—that the synthetic validation in Section 4 does not include; addressing them, together with extending the framework to moving targets, to a rigorously joint (rather than sequential) treatment of self-calibration and image-domain deconvolution, to non-Gaussian or outlier-robust measurement models, and to broader validation geometries, are natural directions for future work.

## Figures and Tables

**Table 1:** Summary of notation introduced in Section 2.

Symbol	Meaning
$p_{tx}$	Exactly known transmitter position
$p_{k}^{0}$, $p_{k}$	Nominal (approximate)/true position of calibration target *k*
$q_{m}^{0}$, $q_{m}$	Nominal (approximate)/true position of receiver *m*
$\delta p_{k}$, $\delta q_{m}$	Small position corrections to target *k*/receiver *m* (unknowns to be estimated)
$\delta t_{tx}$	Transmitter emission-time offset relative to the receiver-1 time reference (unknown)
$\delta t_{m}$	Receiver-*m* clock bias relative to receiver 1 ($\delta t_{1}\equiv 0$ by convention; unknown for m≥2)
$b_{m}=c\;\delta t_{m}$	Clock bias re-expressed as a range-equivalent quantity (Section 3)
$\tau_{km}$	True bistatic propagation delay, transmitter–target *k*–receiver *m*
$\tau_{km}^{0}$	Bistatic delay predicted from nominal (uncorrected) geometry
${\hat{\tau }}_{km}$	Measured time of arrival of target *k*’s echo at receiver *m* (matched-filter/correlation peak)
$\delta {\hat{\tau }}_{km}$	Observed delay residual, ${\hat{\tau }}_{km}-\tau_{km}^{0}$ (a measurement, not an unknown)
$n_{km}$	Measurement noise on ${\hat{\tau }}_{km}$, distinct from the parameter corrections above
${\hat{u}}_{k,tx}$, ${\hat{u}}_{k,m}$	Unit vectors from target *k* toward the transmitter/receiver *m*
x	Stacked unknown-correction vector, $\left[\delta p_{k};\;\delta q_{m};\;\delta t_{tx},\delta t_{m}\right]$
A, b, n	Linearized system matrix, measurement vector, and noise vector of (6)
$W^{-1}$	Measurement noise covariance, Cov(n)
$\Sigma_{0}$	Prior covariance on x (belief about the nominal geometry before any measurement)
J, $J_{\mathrm{MAP}}$	Data-only and Bayesian (MAP) Fisher information matrices, (8), (9)
Q(·)	Matched-filter image score at a candidate point
J(·)	Coherent multistatic image-sharpness objective, (11)

**Table 2:** Effect of bandwidth and windowing on image quality metrics (after self-calibration).

Metric	Single Freq., No Window	81 Freq., 4 GHz, Hamming
Peak-to-mean ratio	13.47	103.06
Correlation with true scene	0.0105	0.3483
Self-calibration sharpness gain (*J*)	1.12×	2.99×

**Table 3:** Dirty (wideband, windowed, self-calibrated) image versus CLEAN-restored image.

Metric	Dirty Image	CLEAN Restored
Correlation with true scene	0.3483	0.2905
Peak-to-mean ratio	103.06	1667.23
Entropy	10.71	6.20

**Table 4:** Target resolvability metrics for the 2-instance eagle scene, before self-calibration, after self-calibration, and after self-calibration plus CLEAN.

Metric	Before Self-Cal	After Self-Cal	+CLEAN
Detected peaks (of 2 true targets)	4	4	4
Targets matched (1 mainlobe)	0	2	2
Mean centroid error [m]	0.136	0.031	0.031
Worst-case centroid error [m]	0.142	0.031	0.038
Resolved (true sep. 4 m)?	No	Yes (4.000 m)	Yes (4.000 m)

## Data Availability

The MATLAB (version R2025b) code used to generate the geometry, diagnostics, and figures described in this manuscript is available from the corresponding author upon reasonable request.
